# Fine Tuning of an Oxidative Stress Model with Sodium Iodate Revealed Protective Effect of NF-κB Inhibition and Sex-Specific Difference in Susceptibility of the Retinal Pigment Epithelium

**DOI:** 10.3390/antiox11010103

**Published:** 2021-12-31

**Authors:** Xue Yang, Usha Rai, Jin-Yong Chung, Noriko Esumi

**Affiliations:** Wilmer Eye Institute, Johns Hopkins University School of Medicine, Smith Building Room 3041, 400 North Broadway, Baltimore, MD 21231, USA; xyangcelia@gmail.com (X.Y.); urai2@jhmi.edu (U.R.); jchung9@jhmi.edu (J.-Y.C.)

**Keywords:** retinal pigment epithelium, sodium iodate, oxidative stress, IKKbeta, NF-kappaB, sex differences, anti-oxidant, catalase

## Abstract

Oxidative stress of the retinal pigment epithelium (RPE) is a major risk factor for age-related macular degeneration (AMD). As a dry AMD model via oxidative stress, sodium iodate (NaIO_3_), which is primarily toxic to the RPE, has often been used at a high dose to cause RPE death for studying photoreceptor degeneration. Thus, characterization of RPE damage by a low dose of NaIO_3_ is still limited. To quantify RPE damage caused by NaIO_3_ in mice, we recently developed a morphometric method using RPE flat-mounts. Here, we report that NaIO_3_ has a narrow range of dose–effect correlation at 11–18 mg/kg body weight in male C57BL/6J mice. We evaluated the usefulness of our quantification method in two experimental settings. First, we tested the effect of NF-κB inhibition on NaIO_3_-induced RPE damage in male C57BL/6J mice. IKKβ inhibitor BAY 651942 suppressed upregulation of NF-κB targets and protected the RPE from oxidative stress. Second, we tested sex-specific differences in NaIO_3_-induced RPE damage in C57BL/6J mice using a low dose near the threshold. NaIO_3_ caused more severe RPE damage in female mice than in male mice. These results demonstrate the usefulness of the quantification method and the importance of fine-tuning of the NaIO_3_ dose. The results also show the therapeutic potential of IKKβ inhibition for oxidative stress-related RPE diseases, and reveal previously-unrecognized sex-specific differences in RPE susceptibility to oxidative stress.

## 1. Introduction

The retinal pigment epithelium (RPE) is a single layer of epithelial cells displaying a cobblestone-like appearance and located between retinal photoreceptor cells and the choroid of the eye [1]. The RPE has multiple critical functions to support photoreceptors, including maintenance of the retinoid cycle, phagocytosis of their outer segments, and providing oxygen and nutrients to them [1]. Without RPE cells, therefore, normal vision cannot be achieved. While RPE cells are post-mitotic and terminally differentiated, they can lose the epithelial integrity and dedifferentiate in pathological conditions [2,3,4]. Oxidative stress is a constant threat to the RPE due to daily phagocytosis of photoreceptor outer segments containing oxidized lipids and its localization next to the dense vascular network of the choroid, resulting in accumulated oxidative damage with age [5,6]. Oxidative stress is regarded as one of the major risk factors for developing age-related macular degeneration (AMD), the leading cause of blindness in the elderly [7,8,9]. In addition, oxidative stress can also cause phenotypic changes of RPE cells resembling the epithelial to mesenchymal transition (EMT) [2,10,11,12].

Sodium iodate (NaIO_3_), an oxidizing agent that is primarily toxic to the RPE, has long been used as a model for in vivo oxidative stress in various mammals in which photoreceptor cell death follows RPE damage [13,14]. In many previous studies, a high dose [40–100 mg/kg body weight (BW)] of NaIO_3_ was used intentionally to destroy the RPE and create secondary retinal (photoreceptor) degeneration for analyses [15,16,17,18,19,20,21,22,23]. Thus, detailed characterization of NaIO_3_-induced RPE damage with a low dose is still limited. With NaIO_3_ at 20 mg/kg BW, a gradient of RPE damage was reported by dividing the area into three regions with distinct RPE morphologies on RPE flat-mounts, i.e., the periphery with polygonal RPE cells, the center with RPE atrophy and loss, and the transitional zone with irregularly-shaped RPE cells between them in both AKR/J and MRL/MpJ mouse strains [24]. In our hands, NaIO_3_ at 20 mg/kg BW resulted in a nearly complete RPE loss in male C57BL/6J mice. Recently, to refine the NaIO_3_-induced RPE damage model, the effects of NaIO_3_ were analyzed in detail with respect to mouse strain, dosage, and time points, and 15 mg/kg BW was the lowest dose to induce reproducible RPE damage with the three distinct regions, i.e., periphery, transitional zone, and center [25]. With NaIO_3_ at 15 mg/kg BW, we also observed the three regions similar to those reported. Regarding these regions as indicators of the degree of RPE damage, we recently developed a morphometric method to quantify them by scanning the RPE flat-mounts on a microscope (manuscript submitted).

In the response to oxidative stress, nuclear factor kappa B (NF-κB), a master regulator of inflammation and immune response, is one of the key signaling pathways controlling stress response [26,27,28]. In addition, NF-κB directly regulates several EMT transcription factors (EMT-TFs) and mesenchymal genes, and therefore, activation of NF-κB can promote EMT [29,30,31,32]. NF-κB is also identified as a key activator of aging-related gene expression programs [33,34,35]. Thus, NF-κB seems to play multiple roles in the NaIO_3_ model. To test the role of NF-κB, inhibitor of NF-κB (IκB) kinase subunit beta (IKBKB, also known as IKKβ) [36,37,38] has been targeted by pharmacological inhibition or genetic ablation [39,40,41,42]. BAY 651942 (initially described as Compound A), a small molecule ATP-competitive inhibitor that selectively targets IKKβ activity, prevented pulmonary inflammation in animal models of asthma, and also suppressed edema formation in the ear caused by chemical inducers or delayed-type hypersensitivity [42]. In addition, BAY 651942 was effective in reducing myocardial injury following acute ischemia-reperfusion injury [40] and in attenuating lipopolysaccharide-induced neurotoxicity to dopamine neurons in a Parkinson’s disease model [41].

Of the numerous studies using NaIO_3_ in vivo, the majority have utilized male animals, with only a handful of female animals being mentioned, or sex of animals was not described. In addition, a high dose of NaIO_3_ used in these studies likely masked any sex difference, if it existed at all, in the susceptibility or response to NaIO_3_ between male and female animals. Due to the guidelines of US National Institutes of Health (NIH, Bethesda, MD, USA), studies of sex differences in various biological systems, including mouse RPE, have been increasingly more common recently [43]. It has been reported that age-matched male and female C57BL/6J mice show no differences in either retinal function assessed by electroretinograms (ERGs) or RPE function assessed by peak phagocytosis of photoreceptor outer segments in normal conditions [44]. However, few studies analyzed sex differences under stress to date, with some exceptions [43].

In this study, we aimed to (i) further optimize our morphometric method to quantify RPE damage caused by NaIO_3_ and (ii) use this method in two experimental settings to evaluate its usefulness. The first setting was to test the effect of NF-κB pathway inhibition on RPE damage caused by NaIO_3_ in mice using BAY 651942. It inhibited upregulation of NF-κB targets, and protected the RPE from oxidative stress. The second setting was to test sex differences in the susceptibility of mouse RPE to NaIO_3_-induced oxidative stress. The RPE of female mice was more susceptible to NaIO_3_ than that of male mice. These two examples of applications show the usefulness of our quantification method. In addition, fine tuning of the NaIO_3_ dose unexpectedly revealed sex-specific differences in RPE susceptibility to oxidative stress in mice.

## 2. Materials and Methods

### 2.1. Animals

All mice were treated in accordance with the Federal Guide for the Care and Use of Laboratory Animals and the guidelines of the Johns Hopkins University Institutional Animal Care and Use Committee (IACUC; approval codes: MO15M230, MO18M238, and MO21M206). For all animal experiments, we used 8–10-week-old C57BL/6J mice (Jackson Laboratory, Bar Harbor, ME, USA). Male mice were used to test the effect of IKKβ inhibitor BAY 651942, and male and female mice were used to test sex differences in the susceptibility of mouse RPE to NaIO_3_-induced oxidative stress.

### 2.2. Injection of NaIO_3_ in Mice

To induce oxidative stress in mice, we injected NaIO_3_ (S4077, MilliporeSigma, St. Louis, MO, USA) in 200 µL of phosphate-buffered saline (PBS) via tail vein at 10–20 mg/kg BW after dilating the veins by putting the mouse’s tail in warm water. The time of NaIO_3_ injection was regarded as 0 h.

### 2.3. Administration of BAY 651942 in Mice

IKKβ inhibitor BAY 651942 (7-[2-(cyclopropylmethoxy)-6-hydroxyphenyl]-5-[(3S)-3-piperidinyl]-1,4-dihydro-2H-pyrido[2,3-d][1,3]oxazin-2-one hydrochloride) was provided by Bayer AG (Wuppertal, Germany) [42]. The compound was formulated in polyethylene glycol 400 (PEG 400)/H_2_O (80/20) for mouse experiments. BAY 651942 (0, 30, or 60 mg/kg BW) was administered to mice by oral gavage 12 and 1 h before and 4, 24, 48, and 72 h after NaIO_3_ injection. Mouse eyes were analyzed 7 days after NaIO_3_ injection.

### 2.4. RPE/Choroid Flat-Mounts (Called RPE Flat-Mounts)

RPE flat-mounts were processed as previously described [11]. Briefly, mouse eyes were dissected at the equator, the cornea and lens were removed, and the retina was carefully peeled off. The remaining eyecups containing the RPE, choroid, and sclera were immediately fixed in 4% paraformaldehyde (PFA) in 0.1 M phosphate buffer for 10 min at room temperature and transferred into PBS. The eyecups were dissected into quarters by four radial cuts from the periphery toward near the optic disc, and blocked in Tris-buffered saline (TBS) containing 0.25% Triton X-100, 10% normal horse serum (Z0610, Vector Laboratories, Burlingame, CA, USA) and 1% bovine serum albumin (BSA; A9647, MilliporeSigma) at room temperature for 1 h. Then, the eyecups were incubated with anti-ZO-1 antibody (1:200; 402200, rabbit polyclonal, Thermo Fisher Scientific, Grand Island, NY, USA) at 4 °C overnight. After washing with TBS at room temperature, anti-rabbit IgG antibody conjugated with Alexa Fluor 488 (1:500; A21206, Thermo Fisher Scientific) was added. The eyecups were washed with TBS and mounted in Fluorescent Mounting Medium (S3023, Dako, Carpinteria, CA, USA), with the RPE side facing up. Images of the RPE flat-mounts were obtained using an LSM 510 inverted laser scanning confocal microscope (Carl Zeiss, Thornwood, NY, USA).

### 2.5. Quantification of Mouse RPE Damage

To assess RPE damage caused by NaIO_3_ in mice, we recently developed a quantitative method based on RPE cell morphologies on RPE flat-mounts with immunofluorescence of ZO-1 (TJP1, tight junction protein 1), which is located at the RPE cell border and therefore outlines RPE cell shapes (manuscript submitted). Briefly, after staining RPE flat-mounts with anti-ZO-1 antibody as described above, we acquired images of the entire flat-mounts using the tiling function of the LSM 510 microscope. We divided RPE damage into three regions (areas) with distinct RPE morphologies that likely reflect the degrees of RPE damage: morphologically normal-appearing RPE (called “normal RPE”) (periphery), elongated RPE (transitional zone), and damaged RPE without recognizable cells (center). Using ImageJ (1.49v, NIH, Bethesda, MD, USA), we measured these three areas by the number of pixels, and calculated the proportion (%) of each area to the entire RPE area.

### 2.6. RNA Extraction from Mouse RPE and Choroid

To prepare RNA from mouse RPE and choroid separately, we previously modified the RNA extraction method reported for only mouse RPE [45] by using two-step extraction with Trizol reagent (15596018, Thermo Fisher Scientific) followed by RNeasy Micro Kit (74004, Qiagen, Valencia, CA, USA) [11]. Briefly, mouse eyes were dissected to remove the cornea, lens, and retina to obtain the RPE/choroid/sclera eyecup. To release RPE cells, the eyecup was incubated in 200 µL of RNAprotect cell reagent (76526, Qiagen) in a microcentrifuge tube at room temperature for 10 min followed by gentle tapping. The choroid/sclera eyecup was transferred to a new tube containing 250 µL of Trizol. The released RPE cells were collected by centrifugation, and 250 µL of Trizol were added to the RPE pellets. The RPE pellets and the choroid/sclera eyecup were homogenized separately using a pestle grinder, and RNA was purified from each tissue by the two-step extraction: (1) extract RNA into the aqueous phase with Trizol and (2) purify RNA from the aqueous phase with RNeasy Micro Kit.

### 2.7. Reverse Transcription-Quantitative PCR (RT-qPCR)

The mRNA levels of selected genes were analyzed by RT-qPCR. For mRNA expression in mouse RPE, total RNA was prepared by the two-step extraction method as described above. First-strand cDNA was synthesized from 200 ng of total RNA with random primers using SuperScript III reverse transcriptase (18080044, Thermo Fisher Scientific), and real-time PCR was performed with gene-specific primers using C1000 Thermal Cycler (Bio-Rad, Hercules, CA, USA). Relative gene expression was calculated using the 2^−ΔΔCt^ method by normalizing with the geometric mean of three reference genes, *Gapdh*, *Hprt*, and *Actb* (or *Rplp0*). Each sample was analyzed in triplicate. All primers used are listed in Supplementary Table S1.

### 2.8. Statistical Analysis

Statistical analyses were performed using Prism 9 (GraphPad Software, La Jolla, CA, USA). The effects of BAY 651942 on mouse RPE damage caused by NaIO_3_ were analyzed by one-way ANOVA. Sex differences in mouse RPE damage caused by NaIO_3_ were analyzed by Student’s *t* test (unpaired, two-tailed). Correlation between RPE damage (%) and gene expression levels and between expression levels of two genes was analyzed by a simple linear regression model. A *p*-value less than 5% (*p* < 0.05) was considered as statistically significant.

## 3. Results

### 3.1. Morphometric Quantification of RPE Damage Revealed a Narrow Range of NaIO_3_ Dose–Effect Correlation in Mice

**Figure 1:**
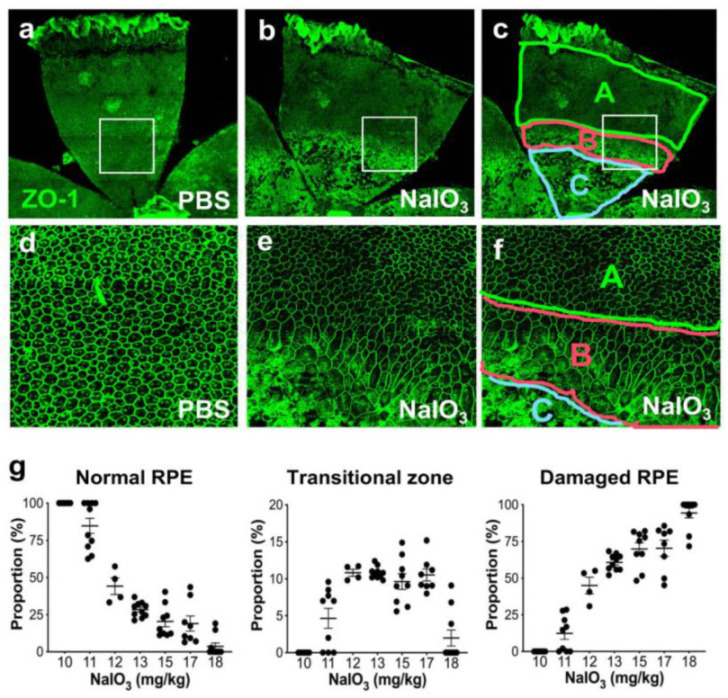
NaIO_3_-induced oxidative stress causes RPE damage with a narrow range of dose–effect correlation in mice. (**a**–**f**) NaIO_3_ causes RPE damage with three distinct morphological regions in mice. Male C57BL/6J mice were injected with vehicle (PBS) (**a**,**d**) or sodium iodate (NaIO_3_) at 15 mg/kg body weight (BW) (**b**,**c**,**e**,**f**), and mouse eyes were analyzed 7 days later by RPE flat-mounts with ZO-1 immunofluorescence (green). One petal representing a quarter of the whole RPE flat-mounts is shown in the top panels (**a**–**c**), with higher magnification images of the squared region in the bottom panels (**d**–**f**). The RPE damage caused by NaIO_3_ was divided into three regions: normal cobblestone-like RPE (A, periphery), elongated and enlarged RPE (B, transitional zone), and severely damaged or lost RPE (C, center) (**c**,**f**). To quantify RPE damage, each region was measured with the number of pixels using ImageJ, and the proportion (%) of each area to the entire RPE was calculated. (**g**) The NaIO_3_ dose range that causes RPE damage in mice. Male C57BL/6J mice were injected with NaIO_3_ at 10–18 mg/kg BW and analyzed 7 days later as described above. NaIO_3_ has a narrow range of dose–effect correlation in causing RPE damage in mice, with no damage at 10 mg/kg BW and nearly complete damage at 18 mg/kg BW. The values represent the means (horizontal lines) and SEM (error bars).

As previously reported [24,25], we also confirmed that RPE damage resulting from NaIO_3_-induced oxidative stress in mice could be divided into three regions with distinct RPE morphologies, i.e., normal-appearing RPE (“normal RPE”) (periphery), elongated RPE (transitional zone), and severely damaged or lost RPE (center) (Figure 1), which seemed to reflect the degrees of RPE damage. Indeed, we observed that the degree of retinal photoreceptor cell death was correlated with these three regions. To assess RPE damage, therefore, we developed a morphometric method to quantify these three regions by scanning the entire RPE flat-mounts with ZO-1 immunofluorescence using the tiling function of a confocal microscope (manuscript submitted).

To better understand the NaIO_3_ model in mice, we tested the effects of different doses of NaIO_3_ on RPE damage using RPE flat-mounts. Since we observed severe RPE damage without recognizable cells in the nearly entire RPE with NaIO_3_ at 20 mg/kg BW in male C57BL/6J mice, we tried NaIO_3_ at 15 mg/kg BW. This low dose produced severe damage in roughly half of the RPE (Figure 1a,b); the three regions were clearly detected (Figure 1c,f). Higher magnification showed the characteristics of each region: normal cobblestone-like RPE (A, periphery), elongated and enlarged RPE cells (B: transitional zone), and degenerated or lost RPE (C: center) (Figure 1d–f). As NaIO_3_ at 10 mg/kg BW did not produce clear RPE damage in male C57BL/6J mice, we wanted to find a threshold and a range of NaIO_3_ dose showing dose dependence. We also wanted to test the sensitivity and reproducibility of our quantification method. Therefore, we injected NaIO_3_ at a dose between 10 and 18 mg/kg BW via tail vein of male C57BL/6J mice and quantified RPE damage 7 days later (Figure 1g). The results showed a narrow window of NaIO_3_ dose–effect correlation from 11 to 18 mg/kg BW, with the threshold producing morphological RPE damage at 11 mg/kg BW. The transitional zone was consistently around 10%, if existed. These results indicate that our quantification method is reproducible and dose-sensitive in this narrow window of NaIO_3_ dose, suggesting that it can be a useful tool for assessing the RPE susceptibility to oxidative stress in mice. Therefore, we employed this method in the two different experimental contexts to test its usefulness, which we describe below.

### 3.2. IKKβ Inhibitor BAY 651942 Protected RPE from NaIO_3_-Induced Oxidative Stress in Mice

The first set of experiments was to examine the effects of NF-κB pathway inhibition on RPE damage caused by NaIO_3_ in mice using BAY 651942, an inhibitor of IKKβ. We first tried to determine an optimal dose of BAY 651942. Since NaIO_3_-induced oxidative stress leads to activation of NF-κB and thereby upregulation of its target genes, we used the suppression of NF-κB target expression as an indicator of BAY 651942 activity. We administered 0 (vehicle), 10, 30, 60, or 100 mg/kg BW of BAY 651942 to male C57BL/6J mice by oral gavage 12 and 1 h before NaIO_3_ (20 mg/kg BW) injection via tail vein, and analyzed the mice 6 h after NaIO_3_ injection. The mRNA levels of four NF-κB target genes, *Icam1*, *Irf1*, *Il1b*, and *Ifnb1*, were analyzed by RT-qPCR, and relative expression was calculated as the ratio to the mRNA level in wild-type mice with neither BAY 651942 nor NaIO_3_ (Figure S1). Although all four genes showed similar dose–effect profiles, only *Icam1* had statistically significant suppression with BAY 651942 at 30 mg/kg BW (*p* = 0.0047) and 60 mg/kg BW (*p* = 0.00005) compared with vehicle control. BAY 651942 at 100 mg/kg BW negated its inhibitory effects on NF-κB likely due to its own toxicity. Based on these results, we decided to use BAY 651942 at 30 and 60 mg/kg BW.

**Figure 2:**
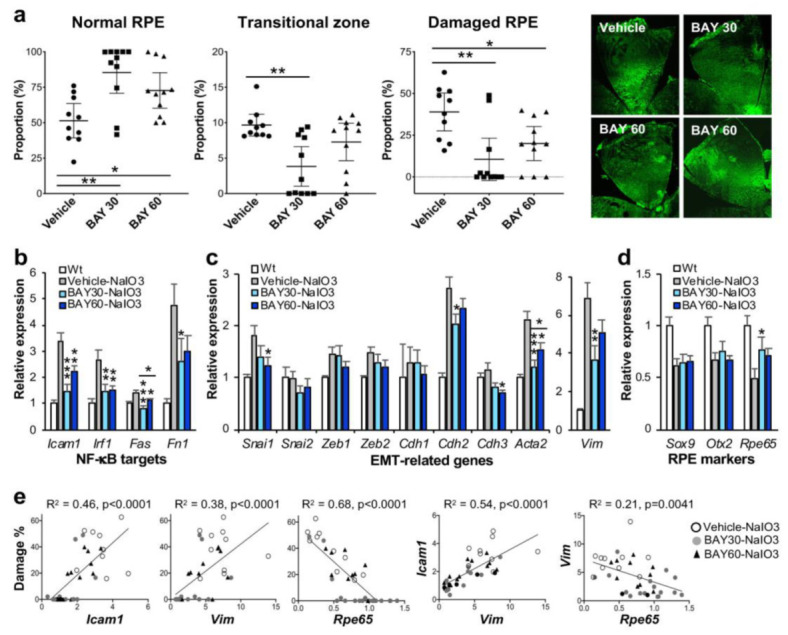
IKKβ inhibitor BAY 651942 protects RPE from NaIO_3_-induced oxidative stress in mice. (**a**) The effects of BAY 651942 on RPE damage caused by NaIO_3_ in mice. Male C57BL/6J mice were given BAY 651942 or vehicle by oral gavage 12 and 1 h before and 4, 24, 48, and 72 h after injection of NaIO_3_ at 16 mg/kg BW via tail vein. Mouse eyes were analyzed 7 days after NaIO_3_ injection as described in Figure 1. The proportions (%) of the three regions, normal RPE (periphery), elongated RPE (transitional zone), and severely damaged RPE (center), were calculated compared to the entire RPE. The results for mice with BAY 651942 at 30 mg/kg BW (BAY 30) or 60 mg/kg BW (BAY 60) were compared with those with vehicle. The values represent the means (horizontal lines) and 95% confidence intervals (error bars). Statistical significance was analyzed by one-way ANOVA and is shown by * *p* < 0.05 and ** *p* < 0.01. On the right, representative images of RPE flat-mounts with ZO-1 immunofluorescence (green) are shown from each group. Two images for BAY 60 are shown, one with no RPE damage (left) and the other with mild damage in the center (right). BAY 651942 significantly protected mouse RPE from NaIO_3_ particularly at 30 mg/kg BW. (**b**–**d**) The effects of BAY 651942 on gene expression changes caused by NaIO_3_ in mice. The contralateral eyes were analyzed by RT-qPCR for expression of genes in three categories: NF-κB targets (**b**), EMT-related factors (**c**), and RPE markers (**d**). Relative expression in the experimental groups, vehicle, BAY 30, and BAY 60, was calculated as the ratio to the expression level in wild-type (Wt) mice. The values represent the means and SEM (error bars). Statistical significance is shown by * *p* < 0.05, ** *p* < 0.01, and *** *p* < 0.001. BAY 651942 significantly inhibited the upregulation of NF-κB targets and EMT markers Acta2 (α-SMA) and Vim (vimentin) compared with vehicle. (**e**) Correlation between RPE damage and gene expression in individual mice. Relative expression of selected genes in (**b**–**d**) were plotted against RPE damage in (**a**) from the same mice and analyzed by linear regression (left 3 panels). Relative expression between two genes was also analyzed by linear regression (right 2 panels). RPE damage was positively correlated with the mRNA level of Icam1 and Vim, and negatively correlated with that of Rpe65.

To test whether BAY 651942 can protect mouse RPE from NaIO_3_-induced oxidative stress, we chose NaIO_3_ at 16 mg/kg BW, a dose within the narrow window that causes more severe RPE damage but not the maximum damage. BAY 651942 was administered to male C57BL/6J mice at 0 (vehicle), 30, and 60 mg/kg BW by oral gavage 12 and 1 h before and 4, 24, 48, and 72 h after injection of NaIO_3_ via tail vein. The mice were euthanized 7 days after NaIO_3_ injection, and the eyes were collected for analyses, one for quantification of RPE damage and the other for gene expression. Using our morphometric method, we measured the three regions, normal RPE (periphery), elongated RPE (transitional zone), and severely damaged RPE (center), as described above. The results showed that BAY 651942 preserved a significantly larger area of normal RPE at both 30 mg/kg BW (*p* = 0.0011) and 60 mg/kg BW (*p* = 0.044) compared with vehicle control (Figure 2a). On the flip side, BAY 651942 significantly reduced RPE damage caused by NaIO_3_ at both 30 mg/kg BW (*p* = 0.0015) and 60 mg/kg BW (*p* = 0.038) compared with vehicle. The mean of transitional zone size was also smaller with BAY 651942 at 30 mg/kg BW (*p* = 0.0023) with the RPE being completely preserved in 5 of 11 mice; however, the transitional zone was still around 10% when clearly existed in 4 mice. Representative images of RPE flat-mounts with ZO-1 immunofluorescence are also presented from each group (Figure 2a, on the right). Single images for vehicle and BAY 651942 at 30 mg/kg BW show moderate and no RPE damage, respectively. Two images for BAY 651942 at 60 mg/kg BW show no damage (left) and mild damage in the center (right). These results indicate that inhibition of the NF-κB pathway can protect mouse RPE from NaIO_3_-induced oxidative stress. The results also suggest that our quantification method of RPE damage in mice can be a sensitive and useful tool.

### 3.3. BAY 651942 Inhibited Upregulation of NF-κB Targets and EMT Markers in the RPE Caused by NaIO_3_ in Mice

The contralateral eyes of the ones used for quantifying RPE damage were analyzed for gene expression using RT-qPCR. We focused on genes in three categories, NF-κB targets, EMT-related factors, and RPE markers. Relative expression in the three experimental groups, BAY 651942 at 0 (vehicle), 30, and 60 mg/kg BW, was calculated as the ratio to the mRNA level in wild-type mice. For NF-κB target genes, *Icam1*, *Irf1*, *Fas*, and *Fn1*, BAY 651942 significantly inhibited their upregulation induced by NaIO_3_ to various degrees as expected (Figure 2b). For EMT-related genes, BAY 651942 did not suppress the mRNA levels of EMT-TFs, *Snai1*, *Snai2*, *Zeb1*, and *Zeb2*, compared with vehicle, except *Snai1* at 60 mg/kg BW (Figure 2c). Since these EMT-TFs are generally upregulated quickly within hours after triggering stimuli such as TGFβ/TNFα treatment and disruption of cell-cell contacts [46], they would return to the baseline by 7 days later, and therefore it was predicted that significant changes of the mRNA levels might not be observed for these genes at this time point. In contrast, the upregulation of EMT markers, *Acta2* (gene for α-smooth muscle actin (α-SMA)) and *Vim* (gene for vimentin), induced by NaIO_3_ was significantly suppressed by BAY 651942 particularly at 30 mg/kg BW compared with vehicle (Figure 2c). Among cadherins that compose adherens junctions, *Cdh1* (gene for E-cadherin), *Cdh2* (N-cadherin), and *Cdh3* (P-cadherin), NaIO_3_ caused upregulation of *Cdh2*, which was suppressed by BAY 651942 at 30 mg/kg BW compared with vehicle. RPE epithelial marker *Cdh3* was slightly decreased with BAY 651942 at 60 mg/kg BW, which likely reflects the EMT-like state suggested by the higher levels of *Acta2* and *Vim* as described above (Figure 2c). Three RPE markers analyzed, *Sox9*, *Otx2*, and *Rpe65*, were all downregulated by NaIO_3_-induced oxidative stress; however, BAY 651942 partially but significantly restored the mRNA levels of *Rpe65* at 30 mg/kg BW (Figure 2d).

Since these gene expression results were mixtures of 10–11 samples for each condition, we wanted to know how RPE damage and gene expression changes were correlated in individual mice. Therefore, we plotted relative expression values of selected genes, *Icam1*, *Vim*, and *Rpe65*, against RPE damage (%) in the same mice individually and analyzed using a linear regression model (Figure 2e, left 3 panels). Morphological RPE damage was positively correlated with mRNA expression levels of *Icam1* and *Vim*, and negatively correlated with those of *Rpe65* in individual samples. We also analyzed correlation between relative expression values of two genes in the same manner using linear regression (Figure 2e, right 2 panels). As predicted, expression levels of *Icam1* and *Vim* were positively correlated, and those of *Vim* and *Rpe65* were negatively correlated. Taken together these results suggest that NaIO_3_-induced oxidative stress trigger the activation of NF-κB, which leads to EMT response and downregulation of *Rpe65*, a critical marker of mature RPE, and ultimately to RPE degeneration if the initial trigger were greater than the level that RPE cells could handle. The results also show that IKKβ inhibitor BAY 651942 can alleviate these events by inhibiting the initial activation of NF-κB signaling.

### 3.4. The Majority of Reported Studies with NaIO_3_ In Vivo Utilized Male Animals

When we characterized knockout (ko) mice (unrelated to this study), we tried to increase the number of wild-type and homozygous ko mice from the same litters by using both males and females. We noticed that RPE damage with a low dose of NaIO_3_ varied widely, and that female mice tended to have more severe RPE damage than male mice. This prompted us to search literatures with the NaIO_3_ model in animals to look for any previous findings of sex differences. Our search in PubMed using the key words “sodium iodate” and “retinal pigment epithelium” yielded 180 hits on 15 November 2021. We collected all available publications written in English, except review articles. After excluding reports with only RPE culture cells, of which the majority were ARPE19 cells, we checked species and sex of animals used in each study. The accumulated studies with NaIO_3_ to date used mostly mice, rats, and rabbits (Table 1). However, when the reports were grouped by published years, it became apparent that most of studies utilized rabbits before 2000, but mice became the choice of animals recently particularly after 2010 (Table S2). As for sex of animals, many reports did not describe it, but when they did, male animals, mice or rats, were used. These data confirmed our speculation that female animals were not sufficiently studied, and therefore sex differences in the susceptibility to NaIO_3_ were still ambiguous.

### 3.5. The RPE Was More Susceptible to NaIO_3_ in Female Mice Than in Male Mice

**Figure 3:**
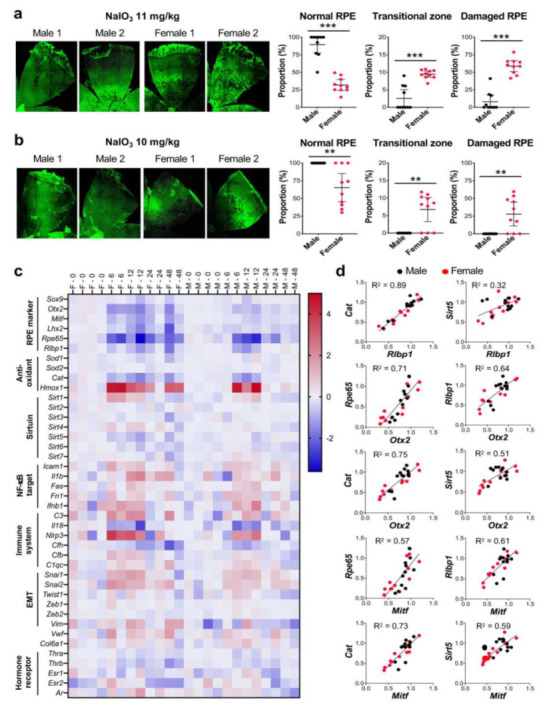
The RPE is more susceptible to NaIO_3_ in female mice than in male mice. (**a**,**b**) Sex-specific differences in the degree of RPE damage caused by NaIO_3_ in mice. Male and female C57BL/6J mice were injected with NaIO_3_ at 11 mg/kg BW (**a**) and 10 mg/kg BW (**b**) via tail vein and analyzed 7 days later, as described in Figure 1. On the left, two representative images of RPE flat-mounts with ZO-1 immunofluorescence (green) are shown for male and female mice with each dose. On the right, the quantification results of all mice in each group are shown. The proportions (%) of the three regions, normal RPE (periphery), elongated RPE (transitional zone), and damaged RPE (center), were calculated compared to the entire RPE. The values represent the means (horizontal lines) and 95% confidence intervals (error bars). The values were compared between male and female mice, and statistical significance was determined by Student’s *t* test (unpaired, two-tailed), which is shown by ** *p* < 0.01 and *** *p* < 0.001. The RPE was more susceptible to NaIO_3_ in female mice than in male mice. (**c**) RPE gene expression changes induced by NaIO_3_ in male and female mice. Male (labeled as M) and female (labeled as F) C57BL/6J mice were injected with NaIO_3_ at 11 mg/kg BW via tail vein, and expression of selected genes in the RPE was analyzed by RT-qPCR at 0, 6, 12, 24, and 48 h after NaIO_3_ injection. Relative expression at each time point was calculated as the ratio to the average of 4 females and 4 males at 0 h and presented as log2. Expression changes of some genes were more profound and/or prolonged in female mice than in male mice. (**d**) Correlation of the expression of selected genes in individual samples. Based on the similar patterns of expression changes revealed in (**c**), correlation of relative expression of Cat and Sirt5 to that of Rlbp1 and two RPE transcription factors Otx2 and Mitf in individual samples was analyzed by linear regression. A strong correlation was observed in the expression levels between Cat and Rlbp1 and between Cat and Otx2 or Mitf.

Based on our literature search described above, we set up the second set of experiments to assess sex differences in the degree of RPE damage caused by NaIO_3_ in mice. We wanted to clarify our earlier impression that NaIO_3_ causes more severe RPE damage in female ko mice than in male ko mice as described above. Male and female C57BL/6J mice were purchased from the Jackson Laboratory, and used for experiments at 8 weeks of age. Based on the NaIO_3_ dose range determined with male mice earlier (Figure 1g), we chose NaIO_3_ at 11 mg/kg BW, the dose at the low-end of the narrow window of dose dependence, and at 10 mg/kg BW, the dose just below the threshold for male mice, to possibly see differences between male and female mice better. NaIO_3_ was injected into mice via tail vein, and the mouse eyes were analyzed 7 days later. We immediately noticed that while male mice showed no or mild RPE damage, female mice suffered from more severe damage with NaIO_3_ at 11 mg/kg BW as representative images indicate (Figure 3a, on the left). Using our quantification method of RPE damage, we measured the three regions, normal RPE (periphery), elongated RPE (transitional zone), and damaged RPE (center). With NaIO_3_ at 11 mg/kg BW, RPE damage was seen in only 4 of 10 male mice, while all 10 female mice showed RPE damage to various degrees (Figure 3a, on the right). The differences between male and female mice were statistically significant for normal RPE (means: males 89.5, females 33.7; *p* < 0.0001), transitional zone (means: males 2.6, females 9.5; *p* < 0.0001), and damaged RPE (means: males 8.0, females 58.8; *p* < 0.0001). With NaIO_3_ at 10 mg/kg BW, none of 10 male mice showed RPE damage, whereas 7 of 10 female mice had RPE damage as shown by representative images (Figure 3b, on the left) and quantification results (Figure 3b, on the right). The differences between male and female mice were statistically significant for normal RPE (means: males 100, females 65.3; *p* = 0.0035), transitional zone (means: males 0, females 6.7; *p* = 0.0016), and damaged RPE (means: males 0, females 27.9; *p* = 0.0044). The transitional zone was consistently around 10% when it was fully present with both NaIO_3_ doses (Figure 3a,b). These results suggest that female mouse RPE is more susceptible to NaIO_3_-induced oxidative stress than male RPE. The results also show that fine tuning of the NaIO_3_ dose near the threshold was key to reveal sex differences in this context.

### 3.6. Expression Changes of Some Genes Caused by NaIO_3_ Were More Profound and Prolonged in the RPE of Female Mice

To gain an insight into the mechanisms of the sex differences found, we analyzed the expression of selected genes in the RPE by RT-qPCR at 0, 6, 12, 24, and 48 h after injection of NaIO_3_ (11 mg/kg BW) in male and female C57BL/6J mice. Relative expression at each time point was calculated as the ratio to the average of four females and four males at 0 h and presented as log2 in a heat map format (Figure 3c). We selected genes in several categories, i.e., RPE markers, anti-oxidants, sirtuins, NF-κB targets, immune system, EMT, and hormone receptors. At a glance, we noticed that expression changes of some genes were more profound or prolonged in female mice compared with male mice. For example, RPE markers were mostly downregulated by NaIO_3_ by 6 h more strongly in females, and then recovered more slowly in females as well. In contrast, although these RPE markers were also downregulated in males, they recovered quickly by 24 h except *Rpe65*. Of anti-oxidant genes analyzed, while *Cat* (catalase) was downregulated (to 30% of control), *Hmox1* (heme oxygenase 1, HO-1) was strongly upregulated by 6 h (25-fold increase). These changes were still detectable at 48 h in female mice, but disappeared by 24 h in males. Most of NF-κB targets and immune-related genes were similarly upregulated in females and males, except *Il1b*, *C3*, and particularly *Nlrp3* that were more strongly upregulated in female RPE. Of EMT-related genes, the expression of *Snai1* and *Snai2* increased in both sexes, but slightly more prominently in females. Interestingly, although changes of *Vim* were modest, it was upregulated in females and downregulated in males. In addition, we noticed that *Cat* and *Rlbp1* had remarkably similar expression changes in females and males, and that *Sirt5* also showed a similar pattern of expression to *Rlbp1* in females, but not in males (Figure 3c). The overall pattern of *Cfh* expression was also similar to that of *Rlbp1*, but to the lesser degree.

Based on these findings, we tested the correlation of expression levels of *Cat*, *Sirt5*, and *Cfh* to those of *Rlbp1* in individual samples by simple linear regression. Since *Rlbp1* encodes a protein that functions in the visual cycle and is regulated by OTX2 along with *Rpe65* [47], we also tested the correlation to the expression levels of *Otx2* and *Mitf* that encode two transcription factors essential for RPE development. Indeed, the mRNA levels of *Rlbp1* were correlated with those of *Cat* (R^2^ = 0.89), *Sirt5* (R^2^ = 0.32), and *Cfh* (R^2^ = 0.71), with the strongest between *Rlbp1* and *Cat* (Figure 3d and Figure S2). The mRNA levels of *Otx2* and *Mitf* were also correlated with those of *Cat* (R^2^ = 0.75 and 0.73, respectively), *Sirt5* (R^2^ = 0.51 and 0.59), and *Cfh* (R^2^ = 0.55 and 0.59). R^2^ values for *Cat* with *Otx2* and *Mitf* were higher than those for *Rpe65* (R^2^ = 0.71 and 0.57) and *Rlbp1* (R^2^ = 0.64 and 0.61) that are known OTX2 targets [47]. These results suggest that *Cat*, and possibly *Sirt5* and *Cfh*, may be regulated by OTX2 and MITF in the RPE.

### 3.7. The Promoter Region of Mouse Cat, Sirt5, and Cfh Contains Canonical OTX2 Binding Sites

To assess the possibility that OTX2 and MITF may regulate *Cat*, *Sirt5*, and *Cfh* in mouse RPE, we looked for binding sites for OTX2 and MITF in the promoter region of these genes. Sequences of the 1 kb 5′-upstream region of mouse *Cat*, *Sirt5*, and *Cfh* were obtained from the UCSC Genome Browser (University of California, Santa Cruz; https://genome.ucsc.edu/ (accessed on 25 March 2021)), and scanned to find canonical OTX2 binding sites (TAATCC/T) [48,49,50] and E-boxes for MITF binding sites (CANNTG, preferably CACGTG, CATGTG, and CACATG) [51]. We found a perfect OTX2 binding site in the promoter region of each gene (Figure S3a). We also found multiple E-boxes in each gene; however, most of them did not have preferred nucleotides at the central positions. We also looked at the 1 kb 5′-upstream region of human *CAT*, *SIRT5*, and *CFH* to find binding sites of these transcription factors. We found a perfect OTX2 binding site in the promoter region of *CAT* and *CFH* (Figure S3b). For *SIRT5*, seven perfect OTX2 sites were present in the first intron. As for MITF binding sites, there were multiple E-boxes in the promoter region of each human gene; however, their central positions were not preferred nucleotides as was the case for mouse genes. These genomic sequence analyses support the speculation that all three genes may be regulated by OTX2, and possibly but less likely by MITF except *Sirt5*/*SIRT5*, in RPE cells.

## 4. Discussion

Oxidative stress plays an important role in both physiology and diseases of the RPE. To study retinal degeneration, NaIO_3_, an oxidizing agent that is exclusively toxic to the RPE, has long been used as an oxidative stress model in vivo in various mammalian species [12,13,14,17,21,24,25,43,52]. However, since most of the earlier studies were conducted with a focus on retinal degeneration by using a high dose of NaIO_3_, understanding of the detailed nature of RPE damage itself with a low dose of NaIO_3_ is still limited [25]. To better understand the NaIO_3_ model in mice, we optimized the dose of NaIO_3_ and further refined a morphometric method to quantify RPE damage caused by NaIO_3_-induced oxidative stress. A caveat of our approach is that RPE damage is analyzed at one specific time point (7 days after NaIO_3_ injection) like a snapshot, which may not accurately reflect the continuously changing nature of RPE damage. It is possible that results may be different if the RPE is analyzed at different time points and that we may just capture differences in the rate of damage progression rather than key differences in the mechanisms causing RPE damage among experimental groups. In spite of these possibilities, however, differences in the degree of RPE damage were detected on day 7, which enables us to explore this method for mechanistic studies to better understand NaIO_3_-induced RPE damage.

In this study, we applied our method for the two experimental purposes to evaluate the usefulness of this method. The first was to test the effects of NF-κB inhibition on RPE damage cause by NaIO_3_ using an IKKβ inhibitor with a higher dose of NaIO_3_ within the narrow window of dose dependence. The second was to test the possibility of sex difference in RPE susceptibility to oxidative stress with the lowest dose of NaIO_3_ within the window of dose–effect correlation. Such fine tuning of the NaIO_3_ dose provided new insights into characteristics of RPE damage resulting from NaIO_3_-induced oxidative stress.

### 4.1. Our Morphometric Method Revealed a Narrow Range of NaIO_3_ Dose–Effect Correlation

After NaIO_3_ injection in mice, we observed three distinct regions of RPE damage. These are the periphery with normal hexagonal cells that have a cobblestone-like appearance, the transitional zone with elongated and enlarged cells that still have ZO-1 at cell junctions, and the center with severely damaged cells that are unrecognizable or lost. Similar observations have been described with NaIO_3_-induced RPE damage in several reports, and therefore these features seem common morphological changes caused by NaIO_3_ [24,25,53]. It should be noted that although we call morphologically normal-appearing RPE as “normal RPE”, normal ZO-1 staining patterns do not indicate that the RPE is physiologically and functionally normal. However, the proportion of these three regions has been quite useful for comparing the degree of overall RPE damage in different experimental conditions for our research projects. In our separate studies, these three regions of RPE damage were correlated well with the extent of photoreceptor cell death assessed by TUNEL assays (manuscript submitted). To estimate the degree of RPE damage, we recently developed a morphometric method to quantify each of these regions on RPE flat-mounts with ZO-1 immunostaining by scanning the entire RPE using the tiling function of a confocal microscope. A similar method has recently been used to quantify the extent of RPE damage by NaIO_3_ in aged DJ-1 knockout (ko) mice [54], S179C-*Timp3* knock-in (ki) mice [55], and anti-thyroid drug-treated mice [53]. In these studies, only the central damage region was measured. In contrast, we quantified all three regions to collect as much information as possible. Interestingly, we found that the transitional zone was consistently around 10% of the entire RPE and showed partial EMT-like characteristics with irregular elongated cells, whose detailed analyses are described elsewhere.

Historically NaIO_3_ has often been used at a high dose to destroy the RPE to study retinal degeneration in animals or to create the condition for cell transplantation studies. Thus, the dose of NaIO_3_ was not optimized or fine-tuned to study RPE damage itself till recently. The majority of earlier studies in mice used NaIO_3_ at a dose higher than 30–50 mg/kg BW, or at least 20 mg/kg BW. However, there are a few studies that utilized a dose lower than 20 mg/kg BW. One study reported that NaIO_3_ at 10 mg/kg BW did not produce RPE damage either morphologically or functionally, and that NaIO_3_ at 20 mg/kg BW was the lowest dose causing RPE damage in 6–8-week-old male C57BL/6J mice [56]. Another study described that while no RPE degeneration was observed with NaIO_3_ at 10 mg/kg BW, 29% of the RPE was degenerated with NaIO_3_ at 15 mg/kg BW in 3-month-old C57BL/6J mice [54]. The RPE was also intact with NaIO_3_ at 10 mg/kg BW in 4-month-old C57BL/6J mice [55]. Most recently, Zhang, et al. analyzed the effects of NaIO_3_ in detail with respect to mouse strain, dosing, and time points, and found 15 mg/kg BW as the lowest dose to induce RPE damage reproducibly [25]. To optimize our experiments, we wanted to define the threshold and a range of NaIO_3_ dose dependence in our hands. With 8–10-week-old male C57BL/6J mice, we found that NaIO_3_ had a narrow window of dose–effect correlation from 11 to 18 mg/kg BW, with no RPE damage at 10 mg/kg BW. In our experience, NaIO_3_ at 20 mg/kg BW consistently caused nearly full RPE damage. Our results clarified for the first time the dose–effect relationship between NaIO_3_ and RPE morphological damage, revealing the narrow range of NaIO_3_ dose dependence and the threshold producing the minimal RPE damage. In this process, our quantification method was useful not only as a reproducible and dose-sensitive tool for assessing the susceptibility of mouse RPE to oxidative stress but also for visualizing the location and degree of RPE damage within the RPE layer.

An important issue that needs to be discussed is the large variability of RPE damage with the same dose of NaIO_3_ or within the same experimental group. We speculate at least two reasons for causing such variability, i.e., technical and biological. As for technical issues, although we are confident in performing tail vein injections, and our success rate is generally above 95%, occasional difficulties and leaks could contribute to the wide-spread outcomes. From biological aspects, we utilized the dose of NaIO_3_ in the range with the steepest changes in dose–effect relationships, particularly the dose at 10–13 mg/kg BW as shown in Figure 1g, and thus inevitable variabilities of in vivo experiments were likely exacerbated. To support this possibility, when we used NaIO_3_ at the dose less than 10 mg/kg BW (always no damage) or more than 20 mg/kg BW (always large severe damage), the results were significantly more consistent within a narrow range but less sensitive to changes of experimental conditions. Accordingly, it is a trade-off between consistency of results and sensitivity to experimental parameters.

### 4.2. Pharmacological Inhibition of NF-κB Signaling Protected the RPE from NaIO_3_-Induced Oxidative Stress in Mice

Considering that NF-κB plays key roles in inflammation, immune reactions, and stress response, including response to oxidative stress [26,27,28], we hypothesized that inhibiting excess NF-κB activation and thereby blocking undesirable stress reactions could protect the RPE against NaIO_3_-induced oxidative stress. Indeed, BAY 651942, a small molecule inhibitor of IKKβ that is an upstream regulator of NF-κB [36,37,38], significantly decreased RPE damage by NaIO_3_, which was clearer at 30 mg/kg than 60 mg/kg BW. We speculate that a high dose of BAY 651942 has some toxic effects as we observed at 100 mg/kg BW in the dose optimization, and therefore the effects of BAY 651942 at 60 mg/kg BW are likely a mixture of beneficial and toxic effects.

The effect of BAY 651942 has been reported in preventing allergen-induced airway and pulmonary inflammation and inhibiting migration of eosinophils and neutrophils in rodents [42]. It also inhibited the release of pro-inflammatory mediator TNFα after a lipopolysaccharide challenge [42]. In our present study, BAY 651942 inhibited upregulation of NF-κB targets and EMT markers caused by NaIO_3_ in mouse RPE as expected. As inflammation plays important roles in various diseases including AMD, blocking NF-κB signaling through inhibition of IKKβ has been tested as a potential strategy for AMD treatment in mouse models. Conditional knockout of *Ikbkb* (also known as *Ikkß* or *Ikk2*) in mouse RPE and retina and TPCA-1, a chemical inhibitor of IKKβ, significantly reduced laser-induced choroidal neovascularization (CNV), an animal model of wet AMD [39,57]. Although NaIO_3_ creates an acute oxidative stress, it has been regarded as a dry AMD inducer because NaIO_3_ primarily causes RPE damage followed by retinal degeneration and thus mimics many features of dry AMD [7,13,14,58]. In our present study, IKKβ inhibitor BAY 651942 significantly protected the RPE from NaIO_3_ in mice. Of note, NF-κB inhibition suppresses *Alu* RNA-induced NLRP3 inflammasome priming in a dry AMD model [59]. Thus, it would be interesting to explore the potential of IKKβ inhibitors for prevention and/or treatment of both dry and wet AMD. NF-κB inhibition could be beneficial at multiple levels in AMD, such as inflammation, complement activation, and angiogenesis.

### 4.3. Many Previous Studies with NaIO_3_ In Vivo Utilized Male Animals

Since NIH announced the requirement of “consideration of sex as a biological variable in NIH-funded research” (NOT-OD-15-102), studies using both male and female animals likely increased, and we have started seeing more reports analyzing both sexes or at least clarifying sex of animals. When we analyzed ko mice (unrelated to this study), we noticed that when we mixed male and female mice in the experiments, the extent of RPE damage with a low dose of NaIO_3_ varied widely, which made us wonder if there might be sex differences in RPE susceptibility to oxidative stress. We wanted to know whether such sex differences had been reported before. Our literature search for animal studies with NaIO_3_ is by no means complete because we focused only on retrievable papers written in English. Nonetheless, our search revealed interesting trends, and confirmed our speculation that RPE damage by NaIO_3_ in vivo has not been much studied in the context of sex differences. While the majority of studies utilized rabbits before 2000, the choice of animals has shifted to mice particularly after 2010. We speculate that this shift might be related, at least in part, to the fact that mice are smaller in size and therefore lower in cost, and that genetically engineered ko and ki mice became available for many genes [54,55,60,61,62,63]. As for sex of animals, many papers did not describe it, but when they did, the majority of animals were males. However, we can expect that sex of animals will be described more often, and more studies will use both male and female animals in the future, thanks to the NIH grant guidelines.

### 4.4. The RPE Was More Susceptible to NaIO_3_ in Female Mice Than in Male Mice

With the NaIO_3_ dose around the threshold, we found that the RPE was more susceptible to NaIO_3_ in female mice than in male mice by quantifying RPE damage. Although there are several previous studies with both male and female animals, some of them utilized a high dose of NaIO_3_ that severely damaged the RPE, and therefore likely masked any sex difference even if it existed [19], and the others did not describe their results with respect to sex differences [53,54,64,65]. In normal conditions, no sex difference was observed in either a- and b-wave amplitudes of ERG or RPE phagocytosis of photoreceptor outer segments between age-matched male and female C57BL/6J mice [44]. Interestingly, it has been reported that ERG c-wave amplitudes were significantly lower in male C57BL/6J mice than in age-matched female mice [66]. However, sex differences under stresses have not been sufficiently studied to date. There is a report showing sex differences in the retina and RPE after NaIO_3_ injection [43]. To investigate the role of systemic inflammation such as rheumatoid arthritis (RA) in AMD, the authors analyzed the effects of collagen-induced arthritis (CIA) on laser-induced CNV as a wet AMD model and NaIO_3_-induced RPE and retinal degeneration as a dry AMD model in C57BL/6J mice. Interestingly, CIA had opposite effects on these AMD models, i.e., CIA reduced the size of CNV but exacerbated NaIO_3_-induced RPE and retinal damage, which is consistent with the epidemiological finding that RA increases the risk for dry AMD, but not for wet AMD [43,67]. They reported that NaIO_3_ caused a greater decrease in retinal thickness in male mice than in female mice, but that sex difference was not observed in ERG c-wave amplitudes or elongated RPE cell shapes caused by NaIO_3_. Importantly, the authors noted that the observed sex difference in disease severity of the NaIO_3_ model in mice is opposite to that seen in human patients because AMD is slightly more prevalent, at least not less, in women than in men [43]. Based on our experience, 50 mg/kg NaIO_3_ used in their study is a high dose that produces severe RPE damage. Therefore, we speculate that it might be difficult to detect sex differences with such a high dose. Our results obtained with the near-threshold dose that the RPE is more susceptible to oxidative stress in female mice than in male mice are compatible with the clinical observation of sex prevalence in AMD patients. Although sex hormones such as estrogen and the estrus cycle in female mice need to be considered [66], our study included two separate sets of experiments using different doses with 10 mice each group, which led to the same conclusion as our unrelated study with ko mice also yielded. To obtain these findings, our quantification method for RPE damage and fine-tuning of the NaIO_3_ dose have been the key.

### 4.5. Expression Changes of Some Genes Were More Profound and Prolonged in Female Mice

To gain any hint for the possible mechanisms for sex differences in RPE susceptibility to oxidative stress, we analyzed the expression of selected genes at different time points after NaIO_3_ injection. Gene expression patterns varied greatly depending on genes in two aspects, i.e., direction of expression changes (increase, decrease, or no change) and sex-dependent differences (present or absent). When expression time-course showed sex differences, expression changes tended to be greater and lasted longer in female mice than in male mice. Although it is currently unclear whether these changes are causative, directly or indirectly, or consequences of a primary event, we find it intriguing that *Cat*, *Sirt5*, and to the lesser degree *Cfh* showed a similar time-course of expression changes to *Rlbp1.* Particularly, the expression changes of *Cat* and *Rlbp1* were nearly identical. The decrease of *Cat* expression that lasted longer in female mice than in male mice drew our attention because it encodes catalase, a critical anti-oxidant enzyme that degrades H_2_O_2_ to H_2_O and O_2_ [68,69]. Therefore, the prolonged low level of catalase may have played a role in producing more severe RPE damage and/or slow recovery in female mice even if it was not a primary event but a downstream effect. The importance of catalase in the RPE for protecting both RPE cells and neighboring photoreceptors from oxidative stress has been demonstrated. Using adenovirus carrying the catalase gene, overexpression of catalase in the RPE markedly reduced oxidative stress markers and significantly protected photoreceptors from cell death in the mouse light damage model [70]. Interestingly, catalase overexpression targeted to the mitochondria increases a life span by 4.5–5.5 months (17–21%) in transgenic mice [71]. Another anti-oxidant gene that stood out was *Hmox1* (protein HO-1), whose mRNA levels increased 25-fold by 6 h after NaIO_3_ injection and lasted longer in female mice than in male mice. Recently, it has been reported that NaIO_3_-induced HO-1 overexpression led to RPE cell death with ferroptosis-associated characteristics such as ferrous ion accumulation and lipid peroxidation, and that knockdown or a chemical inhibitor of HO-1 could inhibit RPE ferroptosis [52]. Although the mechanism of NaIO_3_-induced RPE cell death was previously proposed as necroptosis [65], the above-mentioned and another recent paper suggest that ferroptosis may be the major pathway in oxidative stress-mediated RPE cell death [52,72,73,74]. The actual mechanisms of RPE cell death under stresses likely depend on the type, strength, and duration of the stressors [14], and different cell death pathways may not be mutually exclusive. Clearly, further studies are needed to better understand the mechanisms of RPE death from oxidative stress, which is critically important for developing effective treatments for oxidative stress-related RPE diseases such as AMD.

### 4.6. The Promoter Region of Cat, Sirt5, and Cfh Contains Canonical OTX2 Binding Sites

The expression changes of *Cat* and *Rlbp1* were not only nearly identical, but also similar to those of *Otx2* in both female and male mice. Since *Rlbp1* is an OTX2 target gene [47], we wondered whether *Cat*, and possibly *Sirt5* and *Cfh*, are also OTX2 targets in RPE cells. Although it needs to be biochemically proven, there is at least a perfect OTX2 binding site within the 400 bp upstream region of each gene. Given the presence of OTX2 protein in the nucleus of adult mouse RPE cells [49], there seems a good possibility that OTX2 may bind to these perfect sites. Importantly, *Otx2* was most strongly downregulated after NaIO_3_ injection among the four RPE transcription factor genes, *Sox9*, *Otx2*, *Mitf*, and *Lhx2*. Human *CAT*, *SIRT5*, and *CFH* also contain perfect OTX2 sites, one in the upstream region of *CAT* and *CFH* and seven in the first intron of *SIRT5*. We also looked for potential MITF binding sites; however, E-boxes found did not have preferred nucleotides at the central positions, making it less likely for MITF to bind to these E-box sites [51]. Based on these promoter structures, we speculate that the downregulation of *Cat*, *Sirt5*, and *Cfh* may be a secondary effect of the decreased level of OTX2. Further studies are needed to identify the primary events that lead to the downregulation of *Otx2*. Lastly, while many anti-oxidant genes, including those for Phase I and Phase II detoxification enzymes, are regulated by NRF2, catalase is independent of NRF2, and its transcriptional regulation is not fully understood [75,76]. Therefore, it seems important to elucidate the regulatory mechanisms of catalase in the RPE in future studies.

## 5. Conclusions

We reported two applications of a morphometric method to quantify RPE damage caused by NaIO_3_-induced oxidative stress in mice. We found that NaIO_3_ had a narrow range of dose dependence between 11 and 18 mg/kg BW in male C57BL/6J mice. This finding was useful to design experiments by choosing an optimal dose of NaIO_3_ for obtaining clearer results in the two experimental contexts. First, we observed that inhibition of NF-κB signaling with IKKβ inhibitor BAY 651942 protected the RPE from NaIO_3_ in mice. Second, we unexpectedly found sex-specific difference in RPE susceptibility to oxidative stress, i.e., the RPE was more susceptible to NaIO_3_ in female mice than in male mice. These two examples of applications showed the usefulness of our quantification method, the importance of fine-tuning of the NaIO_3_ dose, and the potential of the old NaIO_3_ model to gain new insights.

## Figures and Tables

**Table 1:** A total number of animal studies with NaIO_3_ grouped by sex of animals. Literatures were searched in the PubMed using the key words “sodium iodate, retinal pigment epithelium” on 15 November 2021. Papers reporting animal studies with sodium iodate (NaIO_3_) were selected, and the information for species and sex of animals was collected. A total number of animal studies grouped by species and sex is shown. The number of animal studies separated by years when they were published is shown in Supplementary Table S2.

Sex	Mice	Rats	Rabbits	Cats	Monkey	Pig	Dog	Sheep	Chicken
Male	35	21	3	0	0	0	0	0	0
Female	5	1	6	0	0	0	0	0	0
Male & Female	10	0	3	0	0	1	0	0	0
No description	24	30	34	4	1	0	1	1	1
Total	74	52	46	4	1	1	1	1	1

## Data Availability

Not applicable.

## Supplementary Materials

The following supporting information can be downloaded at https://www.mdpi.com/article/10.3390/antiox11010103/s1, Table S1: Primer sequences for expression analyses by RT-qPCR; Table S2: The number of animal studies with NaIO_3_ grouped by sex of animals; Figure S1: Dose optimization of IKKβ inhibitor BAY 651942 in mice; Figure S2: Expression levels of *Cfh* also correlate with those of *Rlbp1*, *Otx2*, and *Mitf*; Figure S3: The promoter region of *Cat*, *Sirt5*, and *Cfh* contains canonical OTX2 binding sites.

## Abbreviations

AMD	age-related macular degeneration
ATP	adenosine triphosphate
BW	body weight
EMT	epithelial to mesenchymal transition
EMT-TFs	EMT transcription factors
IKKβ	inhibitor of nuclear factor kappa-B kinase subunit beta
NaIO_3_	sodium iodate
NF-κB	nuclear factor kappa B
PBS	phosphate-buffered saline
PFA	paraformaldehyde
RPE	retinal pigment epithelium
RT-qPCR	reverse transcription-quantitative PCR
TBS	Tris-buffered saline
TJP1	tight junction protein 1
ZO-1	zonula occludens-1
