# Supplementary material for: Fine Tuning of an Oxidative Stress Model with Sodium Iodate Revealed Protective Effect of NF-κB Inhibition and Sex-Specific Difference in Susceptibility of the Retinal Pigment Epithelium
Source: Antioxidants (Basel). 2021 Dec 31;11(1):103. doi: 10.3390/antiox11010103 (PMC8773095; doi:10.3390/antiox11010103)
Supplement: Supplementary file 1 [file antioxidants-11-00103-s001.zip › antioxidants-1514331-supplementary.pdf]

Supplements

Oxidative stress of the retinal pigment epithelium: protective effect of NF-κB inhibition and sex-specific difference in susceptibility

Xue Yang[1#], Usha Rai[1#], Jin-Yong Chung[1], and Noriko Esumi[1]*

[1]Wilmer Eye Institute, Johns Hopkins University School of Medicine, 400 North Broadway, Baltimore, Maryland 21231, USA

[#]Equal contribution

*Corresponding author

Supplementary Table S1
Primer sequences for expression analyses by RT-qPCR

Gene	Species	Primer name	Sequence
	M: mouse	F: forward; R: reverse	
**RPE markers**			
Sox9	M	mSox9-F	GTA CCC GCA TCT GCA CAA C
		mSox9-R	TCC ACG AAG GGT CTC TTC TC
Otx2	M	mOtx2-F	AAA TCA ACT TGC CAG AAT CCA
		mOtx2-R	GGC CTC ACT TTG TTC TGA CC
Mitf	M	mMitf-common-F	CTC TCG AGC GTC GTG CAT GCA GAT
		mMitf-common-R	TTG CAA GGC CGG ATC CAT CAA GCC
Lhx2	M	mLhx2-F	CAG CTT GCG CAA AAG ACC
		mLhx2-R	TAA AAG GTT GCG CCT GAA CT
Rpe65	M	mRpe65-F	TCA GGA GAT ATG TAC TTC CTT TGA CA
		mRpe65-R	TTG TAT GGG GCA GTG TGA CT
Rlbp1	M	mRlbp1-F	TGC AAG TGT GAG AGA CAG CA
		mRlbp1-R	GCC TTT GTC CAG CAG TCA A
**Choroid markers**			
Vwf	M	mVwf-F	TTG CTT CTT ACG CCC ATC TC
		mVwf-R	CCA CGC TCA CAG TGG TTA TAC
Col6a1	M	mCol6a1-F	GGG TCT GGA AGA TGC AGT AAA
		mCol6a1-R	GGT CTG TGG CAA TGA TAC TTA GA
**NF-κB targets**			
Icam1	M	mIcam1-F	CCC ACG CTA CCT CTG CTC
		mIcam1-R	GAT GGA TAC CTG AGC ATC ACC
Fn1	M	mFn1-F	ATG GAC GCA TCA CCT GTA CC
		mFn1-R	CTG AAG CAG GTT TCC TCG GT
Il1b	M	mIl1b-F	GTG GCA GCT ACC TGT GTC TT
		mIl1b-R	ATG AGT CAC AGA GGA TGG GC
Irf1	M	mIrf1-F	CCT GCC AGA CAT CGA GGA AG
		mIrf1-R	CGG CTG GAC TTG GAC TTT CT
Fas	M	mFas-F	ACA TGC TGT GGA TCT GGG C
		mFas-R	CCC GCC TCC TCA GCT TTA AA
Ifnb1	M	mIfnb1-F	CCT GGA GCA GCT GAA TGG AA
		mIfnb1-R	TCT TGG ATG GCA AAG GCA GT
**Inflammation & Immune system**			
C3	M	mC3-F	CTG GCC TCT GGA GTA GAT AGA
		mC3-R	AGT CTT CTT CGG TGT GTG AAA
Il18	M	mIl18-F	GAC AGC CTG TGT TCG AGG AT

		mIl18-R	TGG ATC CAT TTC CTC AAA GG
Nlrp3	M	mNlrp3-F	ATG CTG CTT CGA CAT CTC CT
		mNlrp3-R	AAC CAA TGC GAG ATC CTG AC
Cfh	M	mCfh-F	GAA AAA CCA AAG TGC CGA GA
		mCfh-R	GGA GGT GAT GTC TCC ATT GTC
Cfb	M	mCfb-F	TTG TAT CTG AGC AAG GGA AGA G
		mCfb-R	ATA GCC TTG GGC CTT TGT AG
C1qc	M	mC1qc-F	GGC CTG AAG TCC CTT ACA CC
		mC1qc-R	AGC AGC AGG CAA AGT CCA
**EMT-related genes**			
Snai1	M	mSnai1-F	AAG ATG CAC ATC CGA AGC CA
		mSnai1-R	CAG TGG GAG CAG GAG AAT GG
Snai2	M	mSnai2-F	CGA ACC CAC ACA TTG CCT TG
		mSnai2-R	GTG AGG GCA AGA GAA AGG CT
Twist1	M	mTwist1-F	CAT GTC CGC GTC CCA CTA G
		mTwist1-R	TCC AGC TCC AGA GTC TCT AGA C
Zeb1	M	mZeb1-F	TCA TTT ATC CTG AGG CGC CC
		mZeb1-R	TCA CAA TAC GGG CAG GTG AG
Zeb2	M	mZeb2-F	GGA GAG TGT TGT GGA GCA CA
		mZeb2-R	ATT GTG GTC TGG ATC GTG GC
Vim	M	mVim-F	TGC GCC AGC AGT ATG AAA
		mVim-R	GCC TCA GAG AGG TCA GCA AA
Acta2 (α-SMA)	M	mActa2-F	GGC ATC ATC ACC AAC TGG GA
		mActa2-R	GTT CAG TGG TGC CTC TGT CA
**Epithelial markers**			
Cdh1	M	mCdh1-F	TTG GTG TGG GTC AGG AAA TC
		mCdh1-R	GTG TCC CTC CAA ATC CGA TAC
Cdh2	M	mCdh2-F	CCA TCA TCG CTA TCC TTC TGT G
		mCdh2-R	TTG GCT TGG CGC TCT TTA T
Cdh3	M	mCdh3-F	ATC GTG GGA GGT GAT GAT GG
		mCdh3-R	GTG TTG GTC CTG AGC CTC AA
**Anti-oxidant genes**			
Sod1	M	mSod1-F	CTC AGG AGA GCA TTC CAT CAT T
		mSod1-R	CTC CCA GCA TTT CCA GTC TT
Sod2	M	mSod2-F	CAG ATT GCT GCC TGC TCT AA
		mSod2-R	CTG AAG GTA GTA AGC GTG CTC
Cat	M	mCat-F	GAT GGT AAC TGG GAT CTT GTG G
		mCat-R	GTG GGT TTC TCT TCT GGC TAT G
Hmox1	M	mHmox1-F	ACA GAG GAA CAC AAA GAC CAG

		mHmox1-R	GTG TCT GGG ATG AGC TAG TG

Sirtuins

Sirt1	M	mSirt1-F	ACT CCT CAC TAA TGG CTT TCA TTC
		mSirt1-R	GGT GGA GGA ATT GTT TCT GGT AAT
Sirt2	M	mSirt2-F	CCT CTG ACC CTC TGG AGA CC
		mSirt2-R	AAG ACG CTC CTT TTG GGA AC
Sirt3	M	mSirt3-F	TAC AGG CCC AAT GTC ACT CA
		mSirt3-R	CTT CGA CAG ACC GTG CAT GTA
Sirt4	M	mSirt4-F	GTC GTT TTC TTT GGG GAC AC
		mSirt4-R	AGA ATG GCT ATT GGG AGC TTT T
Sirt5	M	mSirt5-F	AGC AAG ATC TGC CTC ACC AT
		mSirt5-R	GCC TGC CAT TTT CTC CAG TA
Sirt6	M	mSirt6-F	AGG CCG TCT GGT CAT TGT C
		mSirt6-R	GCA CAT CAC CTC ATC CAC GTA
Sirt7	M	mSirt7-F	AGC CTA CCC TCA CCC ACA TG
		mSirt7-R	GGT GGA GCC CAT CAC AGT TC

Hormone receptors

Thra	M	mThra-F	AAG CAA GGT GGA GTG TGG
		mThra-R	GCT CGT CTT TGT CCA GGT AAC
Thrb	M	mThrb-F	AAG TTG CCC ATG TTT TGT GAG
		mThrb-R	CTG CCA TTT CCC CAT TCA AAG
Esr1	M	mEsr1-F	AAC CGC CCA TGA TCT ATT CTG
		mEsr1-R	AGA TTC AAG TCC CCA AAG CC
Esr2	M	mEsr2-F	ACG AAG TAG GAA TGG TCA AGT G
		mEsr2-R	GGT TCT CTT GGC TTT GTT CAG
Ar	M	mAr-F	CGA CTA TTA CTT TCC ACC CCA G
		mAr-R	TGC TGG CAC ATA GAT ACT TCT G

Control genes for qPCR

Gapdh	M	mGapdh-F	TCA ACA GCA ACT CCC ACT CTT CCA
		mGapdh-R	ACC CTG TTG CTG TAG CCG TAT TCA
Hprt1	M	mHprt1-F	TGC CGA GGA TTT GGA AAA AGT G
		mHprt1-R	AGA GGG CCA CAA TGT GAT GG
Actb	M	mActb-F	GGC TGT ATT CCC CTC CAT CG
		mActb-R	GGG GTA CTT CAG GGT CAG GA
Rplp0	M	mRplp0-F	ACT GGT CTA GGA CCC GAG AAG
		mRplp0-R	TCC CAC CTT GTC TCC AGT CT

Supplementary Table S2

The number of animal studies with $NaIO_3$ grouped by sex of animals

Literatures were searched in the PubMed using the key words "sodium iodate, retinal pigment epithelium" on November 15, 2021. The information for species and sex of animals was collected from papers reporting animal studies with sodium iodate ($NaIO_3$). The numbers of animal studies are separated by years of their publication. The total number of animal studies is shown in Table 1 in the main text.

2020 – present

Sex	mice	rats	rabbits	cats	monkey	pig	dog	sheep	chicken
Male	13	4	0	0	0	0	0	0	0
Female	1	0	0	0	0	0	0	0	0
Male & Female	5	0	0	0	0	0	0	0	0
No description	5	1	0	0	0	0	0	0	0
**Total**	**24**	**5**	**0**	**0**	**0**	**0**	**0**	**0**	**0**

2010 – 2019

Sex	mice	rats	rabbits	cats	monkey	pig	dog	sheep	chicken
Male	17	11	0	0	0	0	0	0	0
Female	3	1	0	0	0	0	0	0	0
Male & Female	4	0	1	0	0	1	0	0	0
No description	13	14	2	0	0	0	0	0	0
**Total**	**37**	**26**	**3**	**0**	**0**	**1**	**0**	**0**	**0**

2000 – 2009

Sex	mice	rats	rabbits	cats	monkey	pig	dog	sheep	chicken
Male	5	6	2	0	0	0	0	0	0
Female	0	0	0	0	0	0	0	0	0
Male & Female	1	0	0	0	0	0	0	0	0
No description	3	3	1	0	0	0	0	0	0
**Total**	**9**	**9**	**3**	**0**	**0**	**0**	**0**	**0**	**0**

Until 1999

Sex	mice	rats	rabbits	cats	monkey	pig	dog	sheep	chicken
Male	0	0	1	0	0	0	0	0	0
Female	1	0	6	0	0	0	0	0	0
Male & Female	0	0	2	0	0	0	0	0	0
No description	3	12	31	4	1	0	1	1	1
**Total**	**4**	**12**	**40**	**4**	**1**	**0**	**1**	**1**	**1**

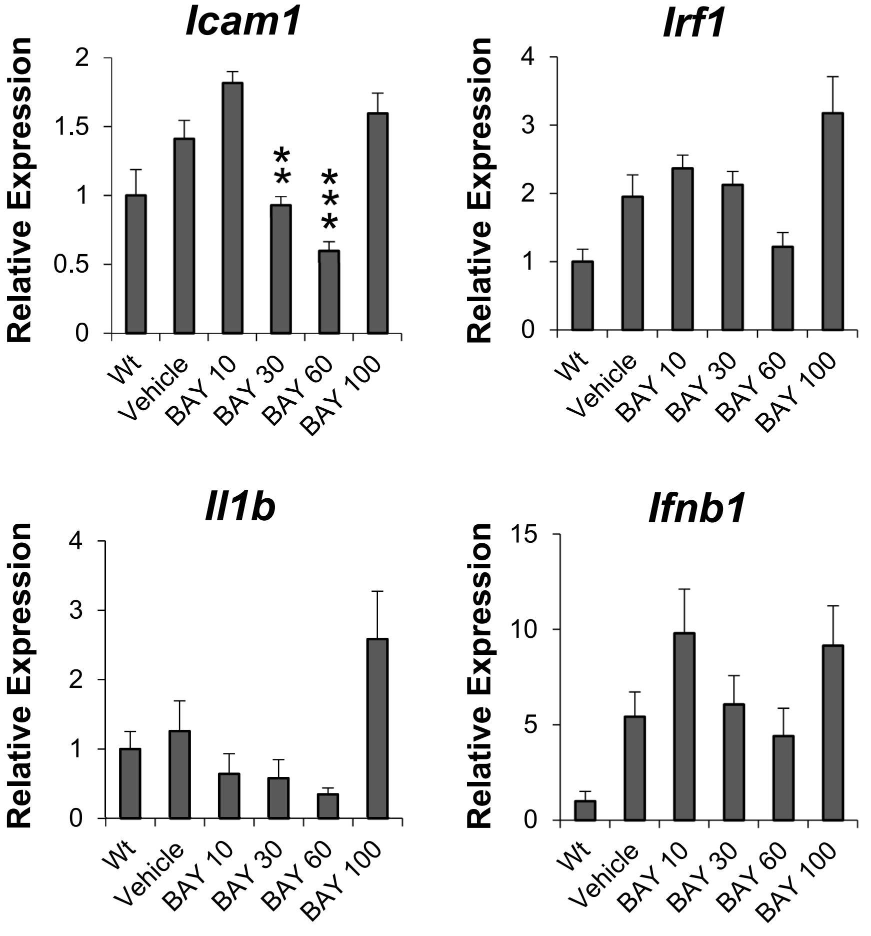

Supplementary Figure S1. Dose optimization of IKKβ inhibitor BAY 651942 in mice. BAY 651942 at 0 (vehicle), 10, 30, 60, and 100 mg/kg body weight (BW) was given to male C57BL/6J mice by oral gavage 12 and 1 h before injection of $NaIO_3$ (20 mg/kg BW) via tail vein. The mRNA levels of NF-κB targets, *Icam1*, *Irf1*, *Il1b*, and *Ifnb1*, in the RPE were analyzed by RT-qPCR to evaluate the efficacy of BAY 651942 as an upstream inhibitor of NF-κB 6 h after $NaIO_3$ injection. Each group had three biological replicates, and each sample was analyzed in triplicate by real-time PCR. Relative expression was calculated as the ratio to the mRNA level in wild-type (Wt) mice without any treatment. The values represent the means and SEM (error bars). Statistical significance was analyzed by one-way ANOVA and is shown by $^{**}p < 0.01$ and $^{***}p < 0.001$. Although all four genes showed similar dose-effect profiles, only *Icam1* showed statistically significant inhibition with BAY 651942 at 30 and 60 mg/kg BW compared with vehicle. BAY 651942 at 100 mg/kg BW lost the inhibitory effects likely due to its own toxicity.

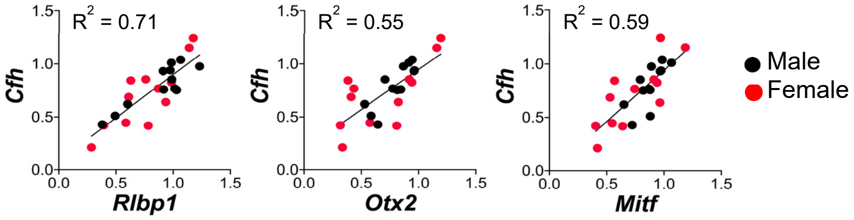

Supplementary Figure S2. Expression levels of *Cfh* also correlate with those of *Rlbp1*, *Otx2*, and *Mitf*. The results in Fig. 3c revealed that *Cfh* also showed $NaIO_3$-induced expression changes similar to those of *Rlbp1*. Therefore, correlation of relative expression of *Cfh* to that of *Rlbp1*, *Otx2*, and *Mitf* in individual samples was analyzed by linear regression as described in Fig. 3d. The expression levels of *Cfh* and the three genes were positively correlated.
